# Influenza-like illness in a Vietnamese province: epidemiology in correlation with weather factors and determinants from the surveillance system

**DOI:** 10.3402/gha.v7.23073

**Published:** 2014-12-08

**Authors:** Dao Thi Minh An, Nguyen Thi Bich Ngoc, Maria Nilsson

**Affiliations:** 1 Institute for Preventive Medicine and Public Health, Hanoi Medical University, Hanoi, Vietnam; 2 Epidemiology and Global Health, Department of Public Health and Clinical Medicine, Umeå University, Umeå, Sweden

**Keywords:** influenza, ILI, epidemiology, time series analysis, ILI surveillance system, weather, climate

## Abstract

**Background:**

Seasonal influenza affects from 5 to 15% of the world's population annually and causes an estimated 250,000–500,000 deaths worldwide. The World Health Organization (WHO) recommends ‘sentinel surveillance’ for influenza-like illness (ILI) because it is simple and calls for standardized methods at a relatively low cost that can be implemented throughout the world. In Vietnam, ILI is a key priority for public health also because of its annually recurring temporal pattern. Two major factors, on which the spread of influenza depends, are the strain of the virus and its rate of mutation, since flu strains constantly mutate as they compete with host immune systems. In the context of global climate change, the role of climatic factors has been discussed, as they may significantly contribute to the cause of large outbreaks of ILI.

**Objectives:**

1) To describe the epidemiology of ILI in Ha Nam province, Vietnam; 2) to seek scientific evidence on the association of ILI occurrence with weather factors in Ha Nam province; and 3) to analyze factors from the Ha Nam ILI surveillance system that contribute to explaining the correlation between the ILI and the weather factors.

**Design:**

A data set of 89,270 monthly reported ILI cases from 2008 to 2012 in Ha Nam was used to describe ILI epidemiological characteristics. Spearman correlation analyses between ILI cases and weather factors were conducted to identify which preceding period of months and weather patterns influenced the occurrence of ILI cases. Ten in-depth interviews with health workers in charge of recording and reporting ILI cases at different levels of the ILI surveillance system were conducted to gain a deeper understanding of factors contributing to explaining the relation between the ILI and the weather factors.

**Results:**

The results indicated that the ILI occurred annually in all districts of the Ha Nam province in the five studied years. An epidemic occurred in 2009 with the number of cases three times higher than the average threshold. There was a relation between the ILI cases in the previous 1 month with ILI cases of the following month. A seasonal cycle of ILI and correlation between weather elements were not clearly detected. A qualitative study showed that the number of ILI cases reported by the Provincial Preventive Medicine Centre (PPMC) in Ha Nam might not have reflected the accurate number of seasonal ILI occurring in this area. This was due to three gaps in the ILI surveillance system that initially were detected through key in-depth interviews in the Duy Tien and Binh Luc districts. They reported inconsistent ways of recording and reporting ILI cases among communes, lack of ILI survey forms, and irregular and delayed feedback from the PPMC.

**Conclusions:**

There were no clear patterns of association between weather factors and ILI cases detected from the five studied years. The number of ILI cases reported by the PPMC in Ha Nam may not reflect adequately the actual number of seasonal ILI occurring in this area due to three weak points in the ILI surveillance system initially detected through the case of the Duy Tien and Binh Luc districts. These three weak points of the system should be examined by a study conducted in the remaining districts in Ha Nam.

According to the US Centre of Disease Control and the European Influenza Surveillance Scheme, ‘Influenza-like illness (ILI), also known as acute respiratory infection (ARI) and flu-like syndrome/symptoms, is a medical diagnosis of possible influenza or other illness causing a set of common symptoms such as fever, shivering, chills, malaise, dry cough, loss of appetite, body aches and nausea, typically in connection with a sudden onset of illness. Common causes of ILI include the common cold and influenza, which tends to be less common but more severe than the common cold’ (1, 2). Any clinical influenza diagnosis is in technical terms an ILI diagnosis. Mostly this is not seen as a problem as most ILI cases regardless of its causes are self-limiting and not severe (3). The 20th century experienced three huge pandemics of influenza, hereinafter called ‘ILI’. The first ILI epidemic caused by A/H1N1 virus occurred in 1918 in Spain and spread all over the world. It affected 20–40% of the worldwide population and 50 million people died (4). The second wave of ILI epidemic occurred in February 1957 and March 1958, which was caused by a new ILI virus (A/H2N2) identified in the Far East (5, 6). The third wave of ILI epidemic occurred in early 1968, caused by an ILI virus detected in Hong Kong, which was similar in some ways to the 1957 pandemic ILI virus (7).

In the 21st century, A/H5N1 ILI first infected humans in 1997 during a poultry outbreak in Hong Kong. From December 12, 2003, to April 12, 2004, the total number of confirmed cases in Thailand and Vietnam combined were 25, of which 19 were fatal (8). In spring 2009, a new ILI virus called ‘A/H1N1/2009’ spread quickly across the United States and throughout the world. There were 74 countries that were affected by the pandemic. CDC estimated that 43–89 million people had H1N1 between April 2009 and April 2010, in which there were approximately 8,870–18,300 H1N1 related deaths (4). Recently, an outbreak of human infections with a new avian influenza A (H7N9) virus was first reported in China by the World Health Organization (WHO) on April 1, 2013 (9).

According to the United Nations Framework Convention on Climate Change, the environment changes with a changing climate, for example, impacting its resilience. Climate change also has different impacts on eco systems and their reproduction and on human health (10). Some scientific studies have indicated the relationship between the ILI prevalence and climatic factors, as well as between ILI cases and climate, with the scale of space and time (11). More specifically, some studies have shown that almost all ILI cases were detected in the summer when the study sites had the greatest rainfall, the highest temperature, and most humidity, revealing that climate factors could be important in the formation of big ILI epidemics (12).

Vietnam is a tropical country experiencing several subtypes of ILI, such as H3N2, H5N1, H1N1, and B. In 2009, a new subtype of ILI, the A/H1N1 virus, appeared in Vietnam, causing an epidemic. This outbreak made the ILI situation in Vietnam more severe and complicated. By May 2010, according to the Ministry of Health, Vietnam had 11,214 cases of ILI A/H1N1/2009 with 58 deaths (13). In the Ha Nam province, an outbreak of A/H3N2 ILI with 200 cases occurred in the industrial zones in 2008 and was followed by a pandemic of A/H1N1, in which many other countries were involved. It occurred in all 116 communes with over 8,000 probable cases in 2009 (14). ILI cases in Ha Nam were reported through routine monthly surveillance reporting of 28 infectious diseases (presented in Annex 1).

This study aims to: 1) describe the epidemiology of ILI in the Ha Nam province, Vietnam; 2) seek scientific evidence on the association of ILI occurrence with weather factors in the Ha Nam province; and 3) analyze factors from the Ha Nam ILI surveillance system that contribute to explaining the correlation between ILI and weather factors.

## Material and methods

### Study area

The Ha Nam province is located in the Red River Delta area, and in 2012, it had a population of about 800,000 people. It is located at the southern gateway of Hanoi and shares borders with six other provinces. The Ha Nam province consists of five districts and one city: Duy Tien, Kim Bang, Binh Luc, Ly Nhan, Thanh Liem, and the city of Phu Ly (herein generally called six study districts). Ha Nam has a tropical monsoon climate. There are two contrasting seasons, which are summer and winter and two transition periods of spring and autumn. The annual average temperature is about 23–24°C, with an average number of hours of sunshine ranging from 1,300 to 1,500 hours/year. The average rainfall is about 1,900 mm and annual average humidity is 85% (15).

### Study design

An initial cross sectional study was conducted in 2008–2012 by reviewing ILI cases reported to the surveillance system of the Preventive Medicine System. The cross-sectional study motivated follow-up questioning, aiming to understand more deeply some of the issues and problems, identified from the quantitative results. Therefore, 10 in-depth interviews were conducted in Ha Nam, consisting of two targeted village health workers, three targeted commune health staff, three targeted district health staff, and two targeted provincial preventive medicine staff. The quantitative study and in-depth interviews were carried out in sequence and were connected.

### Data collection

ILI (International Classification of Disease-10 (ICD-10) with code J10-J11) is categorized in group B of infectious diseases in Vietnam. In the study period, ILI cases were recorded and reported monthly through four levels of the ILI surveillance system, which were from (i) the village level to (ii) the commune level to (iii) the District Health Center (DHC), and finally, to (iv) the Provincial Preventive Medicine Centre (PPMC). Reporting followed a vertical and horizontal system according to the legal provisions on the 28 infectious diseases notifiable in Vietnam (16) as presented in Annex 1. Information on ILI cases were collected through reviewing monthly reporting sheets from 2008 to 2012, which were stored at the PPMC in Ha Nam. Every sheet displayed the monthly aggregated number of ILI cases of all six districts. From these sheets with monthly aggregated ILI data, aggregated ILI cases by district and year were entered into a database, using MS Excel software. Weather data included average temperature (°C), rainfall (mm), relative humidity (%), and sun hours (hours) per month during the period 2008–2012 as recorded by the Centre of Hydrometeorology and Statistics Office of Ha Nam. The monthly aggregated ILI data set was merged with the monthly weather data, for the time series analyses.

The sample in the interview study was purposively selected with the aim of having key informants, who were able to give information to get a deeper understanding of the results identified from the quantitative analysis. Therefore, we approached health workers who were in charge of coordinating and implementing ILI surveillance activities at different levels at definite sites of the ILI surveillance system. The sites and levels selected were those that the quantitative data indicated had problems. Information of these initial interviews would direct the selection of the next persons at different levels for further information. In-depth interviews were carried out until saturation was reached, and there was information enough to suggest a hypothesis for further comprehensive qualitative studies exploring weaknesses in the surveillance system.

A bachelor student, trained and experienced in conducting in-depth interviews and previously coached by senior experienced interviewers, made all of the qualitative data collection for this study as part of her bachelor thesis. The first author of this paper, who was also the supervisor, together with the student used the quantitative data to identify themes that would be raised in the in-depth interviews. An interview guide with themes and questions was prepared and used. The questions were open ended to allow for elaboration and probing by the interviewer. After each interview ended, the student and the supervisor sat together, reviewed the data gathered, discussed issues that needed to be further investigated, and focused for the next interview. In each discussion, the supervisor also helped the student to identify, whom to invite to be interviewed next. This process was implemented for nine rounds and ended when saturation was reached after the tenth interview. From village to province level, all informants gave their informed consent to participate and the interviews were carried out at their work place.

The interviews were recorded with an electronic recorder and on average, each interview lasted 1 hour. The interviewer also took notes by hand in a notebook. The main themes focused on answering problems that were identified from the quantitative study regarding the following issues: 1) practices of health staff recording and reporting ILI cases, 2) the regulation of recording and reporting, 3) other factors of the preventive system that might influence health workers’ practices in identifying, recording and reporting ILI cases from the village- and commune-level to the provincial level, and 4) the mechanism of monitoring and giving feedback from higher levels to their subordinate levels.

### Analysis

In the quantitative analysis, the incidence rate of ILI per 100,000 people was used to describe the distribution of the disease by years and by districts over the period 2008–2012. An np-trend test was performed to examine the tendency of the epidemic from 2008 to 2012 using Stata 10 software (Stata Corporation). An np-trend test is a non-parametric test for trend across ordered groups, developed by Cuzick (18), used here to test whether the rate of ILI increased or decreased significantly through the years, when applying it to longitudinal data. The Analytical Software for Epidemiological Time Series (Epipoi) was used to show the seasonality of ILI cases over years (19). ArcGIS 9.3 was used to map ILI cases by districts in which a spectrum of colors display the average rate of ILI cases/100,000 populations. An auto-correlation (AC) function was estimated to describe the correlation between the ILI cases occurring in the preceding periods of 12 months, hereinafter called ‘lag case’, and ILI cases occurrence of current month using Stata 10.

Spearman correlation analysis was conducted on ILI cases and each of the four weather factors to identify the right preceding periods of months (hereinafter called lag times), which had an influence on the occurrence of ILI cases. The lag times with a correlation between ∣0.3∣ and ∣1∣ were selected to be included in subsequent regression models.

The in-depth interviews were transcribed verbatim. A descriptive content analysis approach focusing on the manifest content in the text was carried out to understand the essence in the data from the interviews.

### Ethical considerations

This study collected monthly aggregated flu cases without having personal information of the cases. Therefore, there was no risk of disclosing any individual information. Feedback of research results to communities in Ha Nam and recommendations from the study to the relevant agencies to take appropriate action to control ILI will be carried out after the completion of this study. In addition, informed consent for the 10 in-depth interviews in the qualitative study was obtained from the key informants.

## Results

**Fig. 1:**
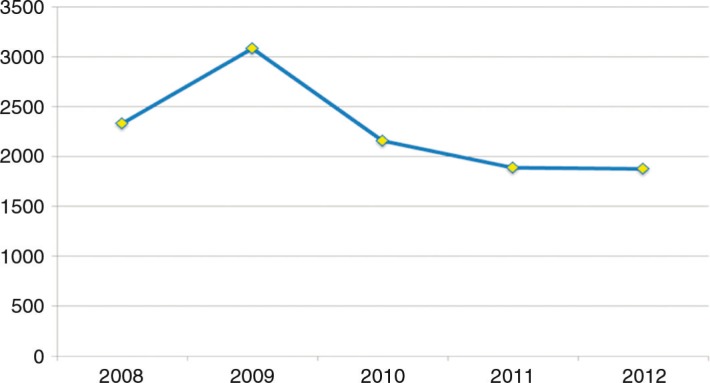
Incidence rate of ILI per 100,000 over the period 2008–2012.

**Fig. 2:**
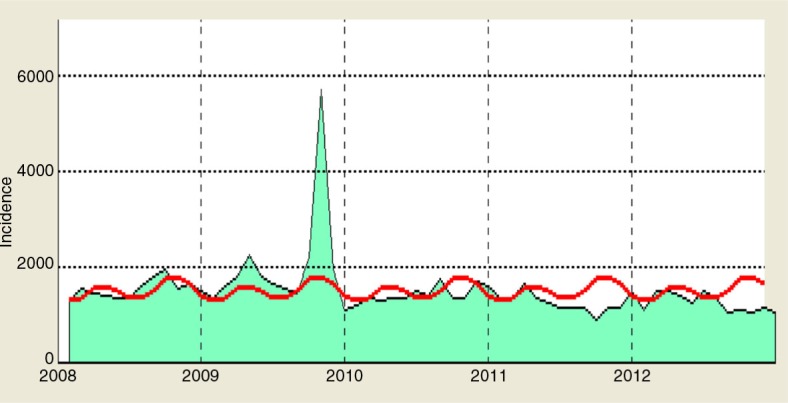
Cycle and seasonality of seasonal ILI from 2008 to 2012. The red line is the average number of ILI cases estimated from 2008 to 2012 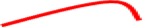. The green area expresses the number of ILI cases over the years 2008–2012 in the Ha Nam province 
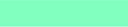
.

**Fig. 3:**
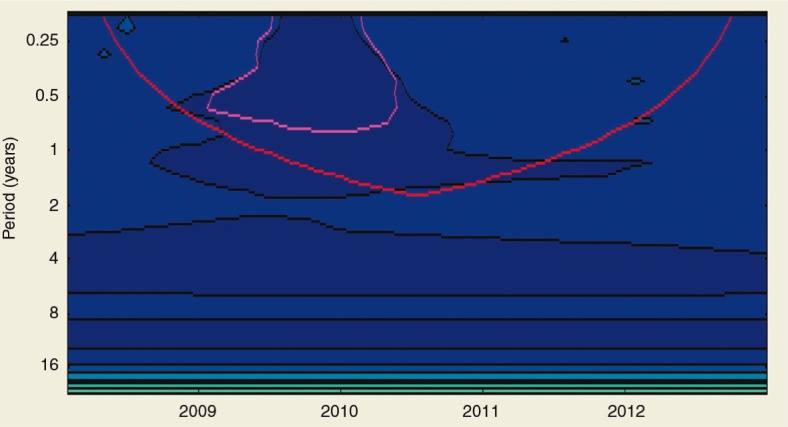
The cyclic pattern of the ILI from 2008 to 2012. The red line limits the statistical significance: 
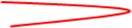
. The pink line is the area of statistical significance: 
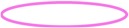
. 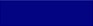 Dark blue color represents the strongest cyclic pattern. 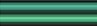 Lighter blue color represents weaker cyclic pattern. Color density represents characteristics of seasonality, of which areas in circled pink displayed the level of statically significance. This figure indicates that the period from 2009 to mid-2010 was statistically significant. However, this period was not enough to show the ILI epidemic.

**Fig. 4:**
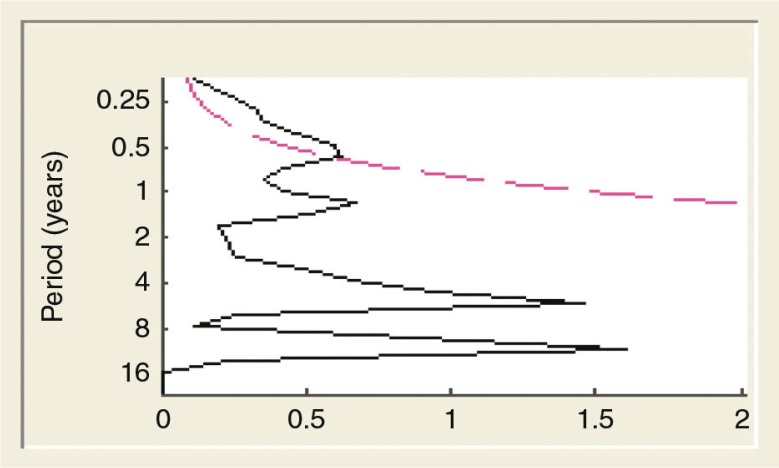
The statistical significance of the flu cycle. The pink line is the area of statistical significance: 
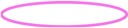
. The back line displays the seasonality of ILI cases: 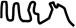. Y-axis displayed period of time (years) for the seasonality of ILI reiterated. X-axis displayed the peak of epidemic after a cycle reiterated. This figure shows four peaks of the ILI corresponding to the period of time of the ILI cycle. The ILI cycle of 1 year once, 4 years once, and 8 years once are very clear but only the cyclic pattern twice a year was significantly statistic (the area lies in the pink line).

**Map 1:**
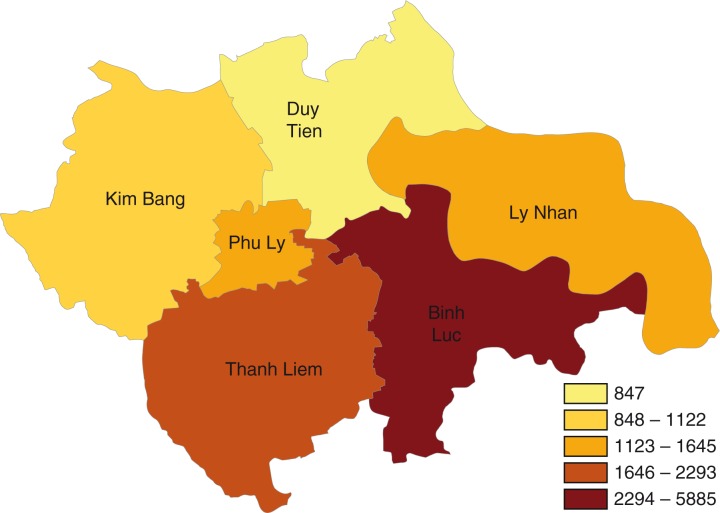
Average incidence rate of ILI per 100,000 people/year in Ha Nam by districts, 2008–2012. Average rate per 100,000 people in 5 years.

The incidence rate of ILI per 100,000 populations ranged from 1,889 to 3,081 annually in the study period. In 2009, the highest rate of ILI was reported, followed by a gradual insignificant decrease in numbers of ILI cases in the years 2010, 2011 and 2012 with *p=*0.07 (Fig. 1). ILI occurred annually and its seasonality was unclear. There were two peaks of ILI cases in 2009, of which the second peak in October was three times higher than the average threshold presented in Fig. 2. The intra-annual cycle of the ILI epidemics occurred twice a year from 2009 until mid-2010 and is presented in Figs. 3 and 4. ILI occurred yearly in all six districts of the Ha Nam province. The average rate of ILI/100,000 people in the period 2008–2012 and over years in the Binh Luc district was always the highest, while the rate of Duy Tien district was always the lowest (Map 1). There was clearly an AC between the number of ILI cases of the previous month with the number of ILI cases of the current month (AC=0.32 with *p<*0.05; presented in Table 1). There were no statistically significant correlations between ILI and temperature, ILI and rainfall, and ILI and hours of sunshine in the study period, except for 2011.

**Fig. 5:**
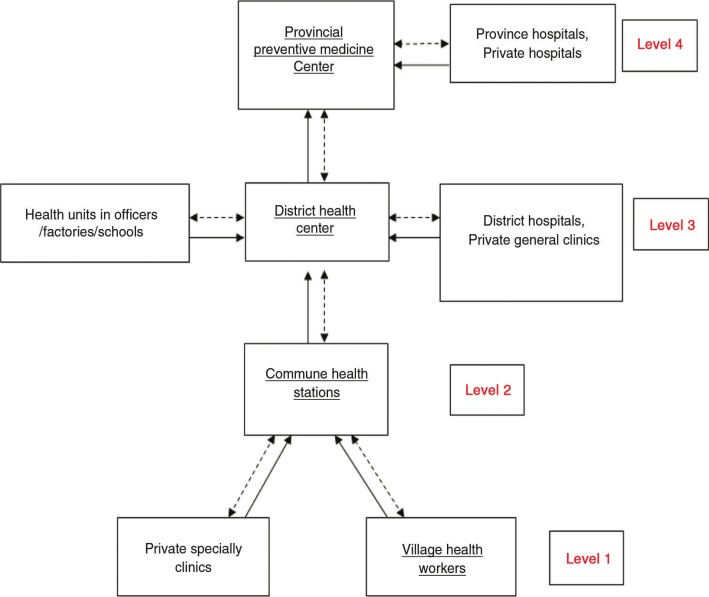
Seasonal ILI surveillance system in Ha Nam province from level of village to province from 2008 to 2012 (17).

**Table 1:** Auto-correlation between ILI cases within 12 lags

LAG	AC	PAC	Q	Prob>Q
1	**0.32**	0.32	6.38	0.01
2	**0.01**	−0.10	6.39	0.04
3	0.02	0.04	6.39	0.09
4	0.04	0.04	6.55	0.16
5	0.06	0.04	6.84	0.23
6	0.14	0.13	8.23	0.22
7	0.09	0.01	8.79	0.26
8	0.03	0.01	8.87	0.35
9	−0.01	−0.03	8.89	0.44
10	0.05	0.07	9.09	0.52
11	0.03	−0.02	9.17	0.60
12	0.02	0.01	9.22	0.68

Note: Values in bold to display the auto-correlation between the number of ILI cases in the previous month and ILI cases in the later month (lag 1, lag 2) with p<0.05. However, this correlation is weak. The remaining values of AC were not statistic significance with p>0.05.

Therefore, the qualitative study focused on understanding if there were obvious reasons, that the stakeholders could reveal, which could potentially explain the finding of no association between weather and occurrence of ILI. It was also of interest to explore why Duy Tien and Binh Luc always had the lowest and the highest number of ILI annually. For example, could there be bias from ILI cases reported that influenced these results? To answer these questions, we conducted a tracking interview, in which we initially interviewed health workers who were responsible for ILI surveillance system at PPMC to understand mechanism of the ILI surveillance system.

The information of interviewers was labeled with numbers below in each quoted sentence (Fig. 5):

^1^A person in a leading position at PPMC in Ha Nam

^2^A person with a leading role in the health staff at PPMC in Ha Nam

^3^A person in the health staff with responsibility in summarizing and reporting ILI cases monthly in the Binh Luc district

^4^A person in a leading role of the CHS in the Duy Tien district

^5^A person in the health staff with responsibility in summarizing and reporting ILI in the Duy Tien district

^6^A VHW in Duy Tien district

^7^A health worker in Binh Luc district

^8^A health station worker in the department of control communicable diseases in DHC of Duy Tien

^9^A person with a leading role at the department of disease control in Binh Luc DHC

^10^A person with a leading role in the department of communicable disease in Duy Tien DHC

### Provincial Preventive Medicine Center

The first key informant^1^ illuminated that:

^1^ILI cases were firstly detected by Village Health Workers (VHWs) or identified by health workers at the Community Health Station (CHSs). CHSs are required to record ILI cases weekly and then report monthly to DHC according to the regulation of reporting infectious diseases. On the fifth every month, CHSs report ILI cases to DHC; on the 10th DHC report aggregated ILI cases to the PPMC and then on the 15th, the PPMC will report aggregated ILI cases to the Preventive Medicine Department, Ministry of Health. Almost all CHSs and DHCs were trained regarding recording and monitoring ILI cases. However, these capacities among districts are different and often depend on the competencies of health workers.

The following informant,^2^ said that:

^2^Capacity of health staff at the DHCs in monitoring ILI cases reported from CHSs depended on leadership, qualification and quality of health staff at each DHC … We ourselves had weak points in capacity of analyzing, interpreting, and giving feedback to the DHC.

When being asked about which level that PPMC staff thought to be the weak link of the ILI surveillance system that might induce bias. The informant^1^ said that:

^1^Communities, where ILI cases from communes were detected is the most important link but still there are issues of concern regarding ILI data.

Therefore, at the next level we decided to conduct an in-depth interview for better understanding of data quality of data at a community level in the Duy Tien and Binh Luc Districts, where quantitative data suggested that there might be problems in recording and reporting ILI cases.

### Commune Health Station (CHS)

In-depth interviews were conducted with the persons who were responsible for 28 infectious diseases surveillance in CHS in Duy Tien and Binh Luc. Through in-depth interviews with these people, we understood that ILI cases were detected, identified, recorded and reported to the CHS by three sources.

The first source was:

^3^People living around the CHS, if they get ill, they primarily visit CHS for being health examined. With ILI cases, based on clinical symptoms such as cough, fever, shortness of breath or epidemiological exposure, we could identify ILI cases at the CHS.

The second source was a network of collaborators called Village Health Workers (VHWs):

^4^Residences staying in the commune such as retired health staff or elderly people having spare time were recruited to work as VHWs. These VHWs were trained to detect and record ILI cases mostly based on clinical symptoms and their individual experiences.

The third source was:

^5^Private health clinics located in the commune, if they had patients with ILI cases (usually severe ones), they have informed the CHSs, but not many of them did that work.

From these three informants at the commune level, it was understood that ILI cases reported by CHSs were underestimated due to many influencing factors as follows:

There were ILI patients of the community but they did not go to CHSs for care.

^3^Not many people came to the CHSs because people would rather go to the district hospital directly. People with mild ILI symptoms might treat themselves without examination at CHS.

VHWs in Binh Luc recorded and reported all ILI cases at any age from three sources

^3^In Binh Luc, we identify all cases of ILI based on clinical symptoms and epidemiology exposure regardless age of patients. We hold a meeting every week with VHWs and they report numbers of ILI cases that they detect during a week. We also call the private clinics to know whether there were cases of ILI identified by these facilities. The aggregated number of ILI cases reported from all three sources were added together then be reported to the district health centre (DHC) on the 5th of each month.

However, health staff in different communes in Duy Tien had recorded and reported ILI cases inconsistently. In the CHS in Trac Van commune, Duy Tien district, health staff only recorded and reported ILI cases of children:

^4^In Duy Tien, only children with ILI were recorded and reported monthly to the DHC. Up until 2012, the PPMC noticed that Duy Tien had reported low numbers of ILI cases and then required our center to record ILI both in adults and children from 2013 upward.

While health staff in Chau Son commune of Duy Tien had recorded and reported ILI cases regardless of ages.

^5^We recorded all ILI cases at all of age.

The length of time that VHWs at Duy Tien and Binh Luc were asked to report ILI cases was quite different as follows:

^3^In the Binh Luc district, the DHC usually send their staff to the CHS to hold a meeting with health staff and VHWs every Thursday.

^4^In Duy Tien that only occurred monthly on the 25th.

### Village health workers (VHWs)

In-depth interviews with VHWs at Binh Luc and Duy Tien districts also indicated that these two districts asked VHWs to report ILI cases in different periods:

^6^In Duy Tien, we often identified ILI symptoms such as cough, fever, running nose, fatigue. Currently, there was no form of recording ILI cases except the form for A/H1N1/09 and A/H5N1. Therefore, any ILI cases identified would be taken notes on in a notebook. Numbers of ILI cases were often required to report on the 25th day every month. To be honest, I am responsible for a village of nearly 1,000 people and surely, I could not contact all people living in the village regularly. When the 25th day is coming, I tried to estimate ILI cases by ask some people living around or the pharmacist whether there were ILI cases occurred. For those ILI cases that were not severe and did not share their illness status with others, we actually could not identify them. The way we recorded ILI was to count numbers of ILI cases identified and add them up to an aggregated number and then report it to the CHS.

^7^In Binh Luc, basic individual information of ILI cases such as name, age, village, symptoms, etc. were noted in a notebook but only aggregated number was reported to the CHS. CHS often holds a meeting with village health workers on Thursday every week. If a VHW was busy and could not be present at the CHS on that day, he or she could delay to report the ILI-cases identified of that week to the following week.

In addition to different practices in recording and reporting ILI among VHWs between Binh Luc and Duy Tien, through the in-depth interviews we also realized that there were factors influencing quality of ILI data collection.

^6^I do this job with 300,000 Vietnamese dong (VND~ 15 USD) allowance per month. This is not enough to live so I have to work on some other jobs and did not much time to work for the CHS.

^7^We were responsible for a large area with a crowded population so there were two VHWs assigned for ILI surveillance in this area but each one only got a half of the monthly allowance (150,000 VND).

### District Health Centre (DHC)

Reasons for the differences in recording and reporting ILI cases between Binh Luc and Duy Tien was raised for discussion in the in-depth interviews with two DHC health staff at Duy Tien and their explanation for the differences as follows:

A health worker, who worked in the department of control communicable diseases in DHC of Duy Tien said that:

^8^VHWs were occupied by many health programs of the CHSs. Sometimes they could not attend the periodical meeting and they asked another person to come instead … Honestly, I can say that VHWs are always changed, old VHWs dropped out, and new VHWs come that make the network of VHW in variation. Duy Tien only reported ILI cases in children. This might be due to some mistakes from receiving ILI form delivered together with immunization form; the new VHWs may think that ILI cases should be identified among children only.

Aggregated numbers of ILI cases were reported to the DHC instead of individual cases and it was confirmed by health staff at the DHC of Binh Luc that:

^9^ILI has no form for recording and reporting, so only aggregated numbers were recorded and reported to DHC while the A/H1N1/2009 investigation did use a form for data collection.

One person in DHC Duy Tien indicated that:

^10^Indeed, we have to accept the aggregated numbers of ILI cases reported from the village and commune, while understanding that without using a form for recording and reporting ILI, biases would occur.

## Discussion

Although this current study indicates that ILI occurred yearly in Ha Nam from 2008 to 2012, seasonality characteristics of ILI cases were not clearly identified. While studies from all over the world showed that ILI epidemics occurred with seasonality characteristics, which marked winter peaks in most countries and regions in the northern hemisphere, such as the United States, Canada and Europe. This current study also indicated that there were characteristics of autocorrelation of ILI among months of the year. That is the correlation of ILI cases of the previous month and the current month with AC1=0.32. According to Johansson, the autocorrelation is a natural element in the process of transmission of infectious diseases and is the relationship between the number of new cases and the number of previous cases (20). A similar result was seen in studies of Soebiyanto et al. (21).

However, this current study does not indicate the relationship between weather factors and ILI, except in 2011, while some studies in the region and elsewhere showed that the spread of ILI was affected partly by weather and climate change (12, 22, 23). Looking back to the original ILI data set reported by Ha Nam preventive medicine department and standard ILI surveillance of the General Preventive Department of Vietnam (Annex 2), it could be seen that, according to this standard, any primary cases with respiratory symptoms and systemic symptoms were to be recorded and reported in order to warn of ILI outbreak. Therefore, these initial cases should receive a laboratory test. If their test results were positive with ILI and, at the same time, there were increasing numbers of similar clinical cases in the community, the epidemiologist would report that there was an ILI outbreak occurring. However, due to limited resources, the system could not test all cases that have suffered from respiratory symptom and systemic symptoms; therefore, after the initial ILI cases are confirmed, if any similar clinical cases occur during that period, they should be recorded, reported and counted as ILI cases without laboratory test. Therefore, the system called ILI surveillance and data extracted from this ILI surveillance could include cases of non-ILI infections and ILI cases. Agents of respiratory infections have differences in seasonality and distribution of transmission pathways. These issues could explain partly the result of there not being an association and no clear pattern of seasonality of ILI in Ha Nam. Besides, the effects of temperature and humidity on ILI transmission may not be equal in temperate and tropical/subtropical climate zones. However, Pham Van Hau et al., in their study of ILI in Vietnam, showed that an increase of the average temperature to 1.5°C may promote the proportion of ILI to 5% (12). Therefore, in-depth interviews conducted in this study helped in interpreting results adding new information.

Results from the in-depth interviews in the current study give initial clues for interpreting this unclear correlation. Firstly, by approaching key persons, who were in charge of conducting and implementing ILI surveillance at different levels of the system, we could identify at which level the weakness of the ILI data collection process mainly originated from. The findings suggested that the community level might have more frequent underestimations of ILI cases at the CHSs, due to several reasons.

The ILI cases surveillance in Ha Nam was passive through the text reports from the level of village and commune. However, the network of VHWs did not appear well-functioning because of the lack of VHWs in terms of quantity and quality. Influencing factors were low wages for VHWs, which forced these VHWs to do extra work for their living and as a result did not dedicate their time for the ILI surveillance work as expected. The backgrounds of VHWs were quite diverse and the variation in this group made the training inefficient. These problems may result in mistakes of Duy Tien such as some VHWs not understanding protocol clearly and only reporting ILI in children. Secondly, the time span for reporting ILI cases was inconsistent between Binh Luc and Duy Tien. Binh Luc asked their VHWs to report ILI cases weekly while Duy Tien asked for monthly ILI reporting. Being asked for a monthly report resulted in VHWs not collecting information regularly on a daily or weekly basis, which may also result in under estimation of ILI cases. Errors in reporting may have occurred consequently because the cases, which happened earlier than the point of reporting time, would have completely recovered and would not be recorded accordingly. In addition, there were ILI cases that were not so weak, did not go to the pharmacists, and/or were informed about their ILI status; therefore, if VHWs did not actively seek this information by going to the commune daily or weekly, they may not get these ILI cases’ information at all.

In Binh Luc, although the CHS asked their VHWs to report ILI cases weekly, some delays still occurred when VHWs were busy at the weekly meeting day and reported ILI cases of previous weeks on the later meetings. Thirdly, almost all ILI cases coming to a private clinic were not recorded and therefore not reported sufficiently to the CHSs.

Although in-depth interviews indicated that community level is the weak point of the ILI surveillance system, it also indicated that some weak points existed at the PPMC and DHC. The case of Duy Tien, that only ILI cases among children were recorded and reported for many years, was realized by DHC in 2012, considering that Duy Tien always had the lowest numbers of ILI cases over the years. This indicates that capacity for analyzing, interpreting, supervising, and giving feedback from the PPMC to the DHCs and from the DHCs to CHSs was very poor.

These issues above were also indicated by findings from a study on ‘Assessment of communicable disease surveillance system and pilot intervention measures’ conducted by Nguyen Thi Phuong Lien in eight provinces/cities in Viet Nam, 2008–2009, with a sample of all health units at commune, district and province level. First, it was indicated that the surveillance system lacked involvement of the network of private health offices and people in communities. Secondly, it was highlighted that the percentage of health units at commune, district and province level having weekly data reported were 26.6, 83.8, and 76.9%, respectively. Data analysis was only conducted in 31.3% at district level and in 62.5% in province level. Feedback information from the district level to CHSs was low at 7.5% while from the province level to DHC it was high at 75%. The study also showed that the quality of surveillance activities was inconsistent between all levels. The rate of units having timely and completed reports was 19.1% in communes and 43.3% at the level of district (25).

Moreover, a big issue regarding forms of recording and reporting ILI cases was also identified from our in-depth interviews. According to the national surveillance system regarding ILI cases report, all PPMCs often submitted the aggregated numbers of ILI cases monthly without any individual information, with the exception when using a survey form when there was a serious epidemic-like A/H1N1/2009 flu. This has resulted in only recording ILI cases as aggregated numbers at community level. According to the WHO and the CDC's regulation of infectious surveillance systems, individual information should be collected sufficiently, especially in the context of emerging variation of ILI in recent years (17). With only aggregated numbers of ILI cases and no form for collecting individual information might have resulted in either overlapping or lacking ILI cases recorded and reported. Without information on age, sex, geography, location, etc., which is considered basic information in a surveillance system, analyses of epidemiological characteristics of ILI cases is neither informative nor effective enough. In comparison with China, a study of Peng Yang et al. illustrated that a surveillance system for ILI and virology data was established in Beijing, from 1 September 2007 to 30 April 2008. This assisted China in the early detection of ILI, defining the distribution of ILI in the community, providing timely information about circulating strains. These data, in turn, can be used to analyze geographical, temporal, and biological differences in circulating ILI strains and assist in monitoring for emerging unusual or critical situations, such as a pandemic (26).

With all of these above issues discussed, it can be understood that the number of ILI cases reported by PPMC in Ha Nam most likely was not adequate and likely under-estimated. As such, it can partially explain why this current study did not report clear correlation between weather factors and ILI cases except in 2011, as well as not finding seasonality of ILI cases in Ha Nam.

This study used the interviews carried out at all three levels (PPMC, DHC, and CHS) as a basis to decide on which level to go deeper in to. This approach helped to reduce redundant interviews and focused more at the community level, which was considered the ‘weakest’ link in the system. Moreover, in this study, we only focused on Duy Tien and Binh Luc districts, where quantitative data suggested having issues that needed to be addressed. The focus, when interviewing key persons who were in charge of coordinating and implementing the ILI surveillance system at the three levels in these two districts, was to build hypotheses as a starting point for future comprehensive studies to be able to later learn and draw conclusions about the whole system. Although the number of in-depth interviews was limited and conducted only in the two districts, trustworthiness and saturation of information was still ensured by using a triangulation approach, in which information from one level was checked by other levels and interviews only ended when there was overlapping and repeated information at different levels and no new information identified.

## Conclusions

The ILI occurred annually in all districts of Ha Nam in 5 years of research. Especially in 2009, the epidemic occurred with the number of cases tripling compared with the average threshold. There was no association between weather factors and ILI cases for the five studied years. Seasonal cycle of ILI and correlation between weather elements and ILI cases were detected without any clear patterns. The number of ILI cases reported by the PPMC in Ha Nam may not reflect the accurate number of seasonal ILI occurring in this area due to some gaps of the ILI surveillance system that were initially detected in the case of Duy Tien and Binh Luc district. Some weak points of the system of ILI surveillance in these two districts were found including: 1) Inconsistent ways of recording and reporting ILI cases between some communes in Duy Tien. In addition, survey forms of ILI cases were not available to analyze the distribution of ILI cases following people's characteristics such as age, sex, immunization status, occupation, etc. 2) The feedback from PPMC in Ha Nam was irregular and delayed.

## Recommendations

It is important to have a good quality system for ILI surveillance, in order to protect public health in the long run. The weak points described in this study need to be further studied in other districts in order to improve the system by getting a comprehensive interpretation of the quality and quantity of ILI cases reported, in relation to the epidemiology of ILI and the scientific evidence of the association between ILI occurrence and weather factors.

## Annex 1

The system of communicable disease surveillance in Viet Nam (17).

## Annex 2

Definition of an ILI case following European Centre for Disease Prevention and Control (24).

The European-CDC ILI case definition requires meeting all of three major criteria: 1) sudden onset of symptoms; 2) at least one of the following four systemic symptoms – fever or feverish, malaise, head-ache, or myalgia; 3) at least one of the following three respiratory tract symptoms – cough, sore throat, or shortness of breath (24).

Definition of an ILI case following General Department of Prevention in Vietnam (17).

✓ Clinical criteria:

At least one of the following four systemic symptoms:

– Fever or feverishness

– Malaise

– Headache

– Myalgia

AND

At least one of the following three respiratory symptoms:

– Cough

– Sore throat

– Shortness of breath

✓ Epidemiological Criteria:

− An epidemiological link by human-to-human transmission

✓ Laboratory Criteria:

At least one the following three:

− Isolation of flu virus from a clinical specimen

− Flu specific antibody response

− Detection of flu virus nucleic acid in a clinical specimen
